# Association between intratumoral free and total VEGF, soluble VEGFR-1, VEGFR-2 and prognosis in breast cancer

**DOI:** 10.1038/sj.bjc.6602374

**Published:** 2005-01-25

**Authors:** H Bando, H A Weich, M Brokelmann, S Horiguchi, N Funata, T Ogawa, M Toi

**Affiliations:** 1 Department of Surgery, Tokyo Metropolitan Komagome Hospital, Tokyo Metropolitan Cancer and Infectious Disease Center, 3-18-22, Honkomagome Bunkyo-ku, Tokyo 113-8677, Japan; 2 Department of Gene Regulation and Differentiation, National Research Centre for Biotechnology, Braunschweig, Germany; 3 Department of Pathology, Tokyo Metropolitan Komagome Hospital, Tokyo Metropolitan Cancer and Infectious Disease Center, 3-18-22, Honkomagome Bunkyo-ku, Tokyo 113-8677, Japan; 4 Department of Clinical Trials and Research, Tokyo Metropolitan Komagome Hospital, Tokyo Metropolitan Cancer and Infectious Disease Center, 3-18-22, Honkomagome Bunkyo-ku, Tokyo 113-8677, Japan

**Keywords:** vascular endothelial growth factor, soluble vascular endothelial growth factor receptor-1, vascular endothelial growth factor receptor-2, neovascularisation, breast cancer

## Abstract

Vascular endothelial growth factor (VEGF) receptors consist of three cell-membrane type receptors (VEGFR-1, VEGFR-2 and VEGFR-3), and soluble form of VEGFR-1 (sVEGFR-1), an intrinsic negative counterpart of the VEGF. In this study, we measured intratumoral protein levels of free and total VEGF, VEGFR-2 and sVEGFR-1 from 202 primary breast cancer tissues and examined their prognostic values. A significant inverse correlation was found between free or total VEGF and oestrogen receptor (ER) status (*P*=0.042 and 0.032, respectively). A univariate analysis showed that low sVEGFR-1 and high total VEGF were significantly associated with poor prognosis in disease-free survival (DFS) and overall survival (OS). The ratio of sVEGFR-1 to total VEGF was a strong prognostic indicator (DFS: *P*=0.008; OS: *P*=0.0002). A multivariate analysis confirmed the independent prognostic values of total VEGF and the ratio of sVEGFR-1 to total VEGF. In subgroup analysis, total VEGF was a significant prognostic indicator for ER-positive tumours but not for ER-negative tumours, whereas sVEGFR-1 was significant for ER-negative tumours but not for ER-positive tumours. In conclusion, the intratumoral sVEGFR-1 level, VEGF level and the ratio of sVEGFR-1 to total VEGF are potent prognostic indicators of primary breast cancer, and might be relevant to ER status.

Vascular endothelial growth factor (VEGF) and its receptors are essential for neovascularisation in cancer. Numerous studies have indicated that intratumoral VEGF expression is significantly correlated with microvessel density and poor prognosis in a variety of human solid cancers including breast cancer, brain tumours, head and neck cancer and gastrointestinal cancer (Toi *et al*, 2001; Ferrara *et al*, 2003). The prognostic value of VEGF has been confirmed not only in immunohistochemical studies but also in other studies using enzyme-linked immunosorbent assay (ELISA) and Northern blotting. In most clinical studies that examined the prognostic value of VEGF in primary breast cancer, intratumoral VEGF expression was a significant marker of poor prognosis in both node-negative and node-positive subgroups (Gasparini, 2000). Thus, these studies concluded that intratumoral VEGF status is an independent prognostic indicator of primary breast cancer.

VEGF binds to two types of cell-membrane receptors, the VEGF receptor (VEGFR)-1 and VEGFR-2 located in the endothelium, and stimulates endothelial migration, proliferation, permeability and survival (Ferrara and Alitalo 1999; Shibuya, 2001). In addition to these two receptors, a soluble form of VEGFR-1 (sVEGFR-1), a naturally occurring and alternatively spliced variant of sVEGFR-1, functions as a high-affinity receptor of VEGF (Kendall and Thomas, 1993; Kendall *et al*, 1996). Since sVEGFR-1 is a secretory protein, it is an intrinsic negative counterpart of VEGF signalling. Recombinant sVEGFR1 binds to all isoforms of VEGF and inhibits VEGF-induced endothelial cell proliferation. Gene therapies involving sVEGFR-1 significantly suppress tumour growth in animal experimental models (Goldman *et al*, 1998; Takayama *et al*, 2000; Mahasreshti *et al*, 2001; Hoshida *et al*, 2002; Sako *et al*, 2004). Kendall *et al* (1996) found that sVEGFR1 is abundant in all identified VEGFR-1 cDNAs from human primary endothelial cells. In our preliminary study using primary breast cancer tissues, sVEGFR1 was frequently coexpressed with VEGF, and the intratumoral balance between sVEGFR1 and VEGF levels showed a significant relationship with survival (Toi *et al*, 2002). According to a recent report on brain tumours, the ratio of sVEGFR-1 to VEGF is significantly decreased in glioblastomas compared with astrocytomas, which indicates the importance of sVEGFR-1 expression in brain tumour growth (Lamszus *et al*, 2003). It is therefore crucial to investigate the relationship between VEGF and its receptors including sVEGFR-1. Previous studies have indicated that intratumoral sVEGFR-1 levels are frequently elevated in human tumour tissues, although the precise upregulation mechanism involving sVEGFR-1 in cancer is largely unknown.

Recently, we have developed a more sensitive sVEGFR-1 ELISA system in addition to new methodologies that permit the separate measurement of free VEGF, which is unbound to the receptors, and total VEGF, which includes both bound and unbound molecules to receptors. In this study, we measured total and free VEGF, sVEGFR-1 and VEGFR-2, as well as Her-2/neu and thymidine phosphorylase (TP) levels in breast tumour cytosols quantitatively and then evaluated those prognostic values. The information from this study is useful not only for assessing the prognostic value of these markers but also for considering the clinical implications of future anti-VEGF therapies paradigm.

## MATERIALS AND METHODS

### Patient population

We randomly selected tissues from 202 patients with operable primary breast cancer who underwent modified radical or partial mastectomy with full dissection of their axillary lymph nodes at the Tokyo Metropolitan Komagome Hospital from 1996 to 1999 with an average follow-up period of 64.0 months and a range of 1.3–93.2 months. Representative samples of the tumour specimens were immediately frozen in liquid nitrogen after surgical resection and stored at −80°C until preparation for ELISA. Pathological examinations were performed on formalin-fixed, paraffin-embedded specimens. The main characteristics of the patients and adjuvant hormone and chemotherapy details are described in Table 1. All patients signed an informed consent according to a protocol approved by the ethics committee of the hospital.

#### Adjuvant therapy and patient follow-up

Indications for and the schedule of adjuvant treatment were decided based on the patient characteristics including axillary nodal involvement (n), tumour size (T), age and oestrogen receptor (ER). Polychemotherapy including six cycles of CA(E)F (cyclophosphamide, adriamycin/epirubicin and 5-fluorouracil (5-FU)) was given to node-positive patients under the age of 60 years, and FU derivatives were given to the remaining node-positive and high-risk node-negative patients. Tamoxifen was given to hormone receptor-positive patients without a history of thrombosis or liver dysfunction for 5 years and additional LH-RH agonist therapy was undertaken for 2 years for premenopausal cases. The patients received radiation to the remaining breast if partial mastectomy was performed, and to the chest wall and draining lymph nodes if more than four lymph nodes were involved. Post-treatment surveillance was carried out according to general practice for breast cancer patients at our institute. Briefly, for the first 5 years physical examinations, haematology and blood chemistry analyses were performed every 3 months, chest X-rays were taken every 6 months and mammography was performed annually. Thereafter, physical, blood and chest X-ray examinations were performed every 6–12 months, and annual mammography was continued. If tumour relapse was suspected, the patient underwent intensive work-up including chest/abdominal computed tomography scans, isotopic bone scans, bone radiography or histological examination. Survival analysis was performed on 186 cases excluding six patients with ductal carcinoma *in situ* (DCIS) and 10 patients who did not show up for the follow-up. The outcomes examined included overall survival (OS) and disease-free survival (DFS), which were calculated from the date of surgery. Overall survival was calculated from the date of surgery to last contact for living patients. Disease-free survival was defined as the period from the date of surgery to the confirmed tumour relapse date for relapsed patients and from the date of surgery to the date of the last follow-up for disease-free patients.

### Histopathologic analysis

Representative sections from all primary tumours were reviewed and analysed by pathologists. The special morphologic features examined included grade, lymph vessel/blood vessel involvement and the number of lymph nodes involved.

#### Sample preparation

Breast tumour tissue samples were treated with two different types of lysis buffer. For the measurement of sVEGFR-1 and TP protein, tissue samples were homogenised in a solution of 10 mM Tris-HCl buffer (pH 7.4) containing 15 mM NaCl, 1.5 mM MgCl_2_, 50 *μ*M potassium phosphate and a protease-inhibitor cocktail. For all other ELISA measurements, samples were individually homogenised in a 10-fold volume of RIPA buffer (0.1% SDS, 1% Tween 20, 0.5% Na-deoxycholate, protease-inhibitor cocktail in phosphate-buffered saline, pH 7.4) and then centrifuged at 14 000 **g** for 20 min. The supernatants were then stored at −80°C until use. A portion of each supernatant was used for protein concentration measurement according to standard protocols (BCA assay, Pierce, Rockford, IL, USA).

### Enzyme-linked immunosorbent assay

Total VEGF protein concentrations in the tumour cytosols were measured using VEGF ELISA kits (R&D Systems, Minneapolis, MN, USA). The measurements were conducted according to the methods recommended by the manufacturer. The minimal detection limit for total VEGF was 31 pg ml^−1^.

A receptor–ligand detection assay was applied to detect free bioactive VEGF following the basic protocol for total VEGF ELISA, except that plates were coated with 0.5 *μ*g ml^−1^ sVEGFR-1 (D1–D6) produced in insect cells (Hornig *et al*, 1999). This ensured that no VEGFR-1 complex forms were recognised. For detection, biotinylated anti-VEGF antibody (R&D Systems, Minneapolis, MN, USA) was used. The minimal detection limit for free VEGF was 20 pg ml^−1^.

Enzyme-linked immunosorbent assay for sVEGFR-1 was performed as previously reported with modifications to improve sensitivity (Toi *et al*, 2002). A human sVEGFR-1 ELISA kit (Bender MedSystems, Vienna, Austria) was used according to the manufacturer's protocol. The minimum detection limit was 100 pg ml^−1^.

The VEGFR-2 protein concentration in tumour lysates was measured using VEGFR-2 ELISA kits (R&D Systems, Minneapolis, MN, USA). The measurements were conducted according to the methods recommended by the manufacturer. The minimal detection limit for VEGFR-2 was 78 pg ml^−1^.

Her-2/neu was determined using a Her-2/neu (c-erbB-2) sandwich enzyme immunoassay (Oncogene Science, Cambridge, MA, USA), which employs a mouse monoclonal antibody for capture and a different biotinylated mouse monoclonal antibody for the detection of human neu protein. The capture and detector reagents specifically bind to the extracellular domain of the neu protein. The minimal detection limit for Her-2/neu was 24 pg ml^−1^.

Thymidine phosphorylase levels were also determined by a colorimetric ELISA. This sandwich immunoassay used two anti-human TP monoclonal antibodies (Nippon Roche Research Center, Kamakura, Japan; 104B and 232-2). The minimal detectable concentration was 1.25 ng ml^−1^.

Levels of ER and progesterone receptor (PgR) were determined using enzyme immunoassay systems from the Otsuka Assay Institute (Tokushima, Japan) as previously reported. The cutoff value of enzyme immunoassay for ER and PgR was 10 fmol mg^−1^ protein.

All protein level measurements made by ELISA were performed in duplicate.

### Statistical methods

The correlation between two factors was evaluated using the Spearman's correlation coefficient by rank and unpaired groups were compared using the Student's *t*-test. Univariate and multivariate Cox regression analyses were carried out to assess potential prognostic indicators of DFS and OS. These features included ER and PgR status, tumour grade (low *vs* intermediate and high grade), tumour size (5 *vs* >5 cm), axillary lymph node involvement (positive *vs* negative), lymph vessel involvement (positive *vs* negative), blood vessel involvement (positive *vs* negative), total VEGF protein concentration (<mean *vs* >mean), free VEGF protein concentration (<mean *vs* >mean), sVEGFR-1 protein concentration (<0.435 *vs* >0.435 ng mg^−1^ protein), VEGFR-2 protein concentration (mean *vs* >mean), Her-2/neu protein concentration (<mean *vs* >mean), TP protein concentration (mean *vs* >mean) and sVEGFR-1/total VEGF (<0.5 *vs* ⩾0.5). All clinical and biological parameters regardless of whether they were statistically significant as seen by the univariate analysis were included in the multivariate analysis. Variables that exhibited statistically significant effects were then retained and the others were dropped.

Multivariate analysis resulted in a final model of five prognostic variables for DFS and four prognostic variables for OS. Models were then generated based on the presence or absence of these variables and constructed to assess the relative risk for relapse and death.

Standard Kaplan–Meier and Cox regression methods were applied for survival analysis using the StatView statistical software Version 5.0 (SAS Institute, Cary, NC, USA). All significance testing was two-sided, where log-rank statistics and Wald statistics were used for univariate and multivariate analysis, respectively. Differences for *P*<0.05 were considered to be statistically significant. The last follow-up date was 31st March 2004.

## RESULTS

### Patient characteristics

The patient characteristics are listed in Table 1. The median age at diagnosis was 55 years with a range of 30–86 years. Five patients who presented with DCIS were excluded from the survival analysis. A total of 110 (54%) underwent adjuvant chemotherapy, and 64% of the patients with positive receptor underwent adjuvant hormonal therapy. In all, 66% of the tumours were ER positive and/or PgR positive, and 81% were intermediate or high-grade tumours.

The protein concentrations of total VEGF, free VEGF, sVEGFR-1, VEGFR-2, TP and Her-2/neu in breast tumour tissue extracts determined by ELISA are listed in Table 2. The correlations between each factor and clinico-pathological parameters were analysed. Total and free VEGF levels were significantly higher in ER-negative tumours, and free VEGF levels were also higher in PgR-negative tumours. Soluble VEGFR-1 levels were higher in PgR-negative tumours, and VEGFR-2 showed no statistically significant correlation with any of the clinico-pathological parameters. Her-2/neu was associated with a larger tumour size, ER negativity and high nuclear grade (*P*=0.01, 0.04 and 0.03, respectively). There was a significant correlation of the protein levels between total VEGF and free VEGF (*P*<0.001, *ρ*=0.905), total VEGF and sVEGFR-1 (*P*<0.001, *ρ*=0.278), free VEGF and sVEGFR-1 (*P*<0.001, *ρ*=0.251), sVEGFR-1 and VEGFR-2 (*P*=0.008, *ρ*=0.190), total VEGF and Her-2/neu (*P*=0.029, *ρ*=0.157), free VEGF and Her-2/neu (*P*=0.04, *ρ*=0.145), total VEGF and TP (*P*=0.004, *ρ*=0.207), free VEGF and TP (*P*=0.017, *ρ*=0.171) and sVEGFR-1 and TP (*P*<0.001, *ρ*=0.270), but none was seen between VEGF and VEGFR-2 by Spearman's rank correlation test (data not shown).

To assess the prognostic value of sVEGFR-1 and the ratio of sVEGFR-1 to total VEGF, we determined the cutoff level according to a stepwise method that gives the optimal separation between a low and high risk of relapse as previously described (Toi *et al*, 2002). The cutoff value for sVEGFR-1 was 0.435 ng mg^−1^ protein, which identified 15.6% of the patients enrolled in the survival analysis as having low sVEGFR-1. For total and free VEGF, VEGFR-2, TP and Her-2/neu, the cutoff values were determined as their respective mean values.

According to the combination analysis, the ratio of sVEGFR-1 to total VEGF concentration (S/V ratio) and its prognostic value were assessed using a similar stepwise separation method as described above. Tumours with an associated S/V ratio of 3.0 were considered to be borderline for achieving a prognostic value for survival analysis. A value of 0.5 was decided on as the cutoff value, with 8.6% of the patients having a low S/V ratio with the most unfavourable prognosis (Table 3).

**Figure 1:**
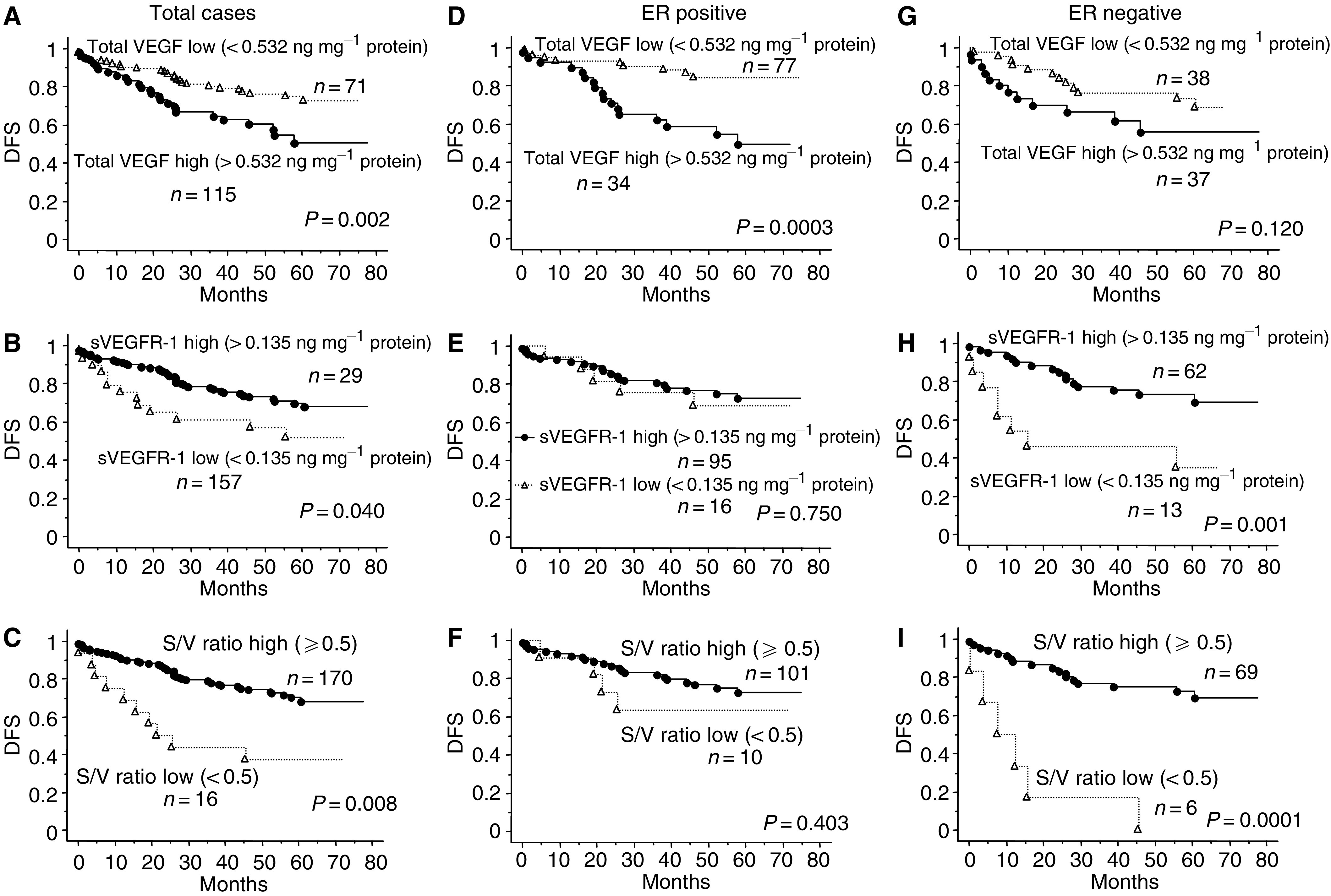
Kaplan–Meier curves for DFS in patients with primary breast cancer by biological markers. Kaplan–Meier curves for DFS in patients with primary breast cancer by biological markers. Disease free survival in the total cases (*n*=186) by VEGF level (**A**), sVEGFR-1 level (**B**) and sVEGFR-1/totalVEGF ratio (S/V ratio; **C**). Disease free survival in the ER-positive cases (*n*=111) by VEGF level (**D**), sVEGFR-1 level (**E**) and S/V ratio (**F**): Disease free survival in the ER-negative cases (*n*=75) by VEGF level (**G**), sVEGFR-1 level (**H**) and S/V ratio (**I**). Levels of VEGF protein more than 0.532 ng mg^−1^ total protein were considered high (solid line) and less than 0.532 ng mg^−1^ total protein negative (dotted line) in (**A**, **D** and **G**). Soluble VEGFR-1 protein levels more than 0.135 ng mg^−1^ total protein were considered high (solid line) and less than 0.135 ng mg^−1^ total protein negative (dotted line) in (**B**, **E** and **H**). S/V ratio more than 0.5 was considered high (solid line) and less than 0.5 negative (dotted line) in (**C**, **F** and **I**). (**A**) The hazard ratio (HR)=2.23 (95% confidence interval (CI)=1.31–3.81, *P*=0.002 using the log-rank test) in favour of total VEGF-low group. (**B**) HR=0.526 (95% CI=0.28–0.98, *P*=0.04 using the log-rank test) in favour of total sVEGFR-1-high group. (**C**) HR=0.368 (95% CI=0.19–0.73, *P*=0.008 using the log-rank test) in favour of S/V ratio-high group. (**D**) In ER-positive subgroup, HR=3.800 (95% CI=1.74–8.31, *P*=0.0003 using the log-rank test) in favour of total VEGF-low group. (**H**) In ER-negative subgroup, HR=0.269 (95% CI=0.11–0.53, *P*=0.001 using the log-rank test) in favour of sVEGFR-1-high group. (**I**) In ER-negative subgroup, HR=0.114 (95% CI=0.04–0.30, *P*=0.0001 using the log-rank test) in favour of sVEGFR-1-high group.

For univariate analyses, patients with low-grade tumours (*P*=0.002), tumours less than 5 cm in size (*P*=0.0001), no lymph node involvement (*P*=0.0001), less vessel involvement (*P*=0.01–0.001), low total VEGF level (*P*=0.002), low free VEGF level (*P*=0.047), high sVEGFR-1 level (*P*=0.04) and a high S/V ratio (*P*=0.008) experienced favourable DFS (Table 3). Overall survival was favourable for patients with negative PgR status (*P*=0.017), low-grade tumours (*P*=0.011), tumours less than 5 cm in size (*P*=0.0002), no lymph node involvement (*P*=0.0005), less vessel involvement (*P*=0.05–0.008), low total VEGF level (*P*=0.006), high sVEGFR-1 level (*P*=0.05) and a high S/V ratio (*P*=0.0002). Figure 1 shows DFS curves of the tumour-related prognostic features total VEGF and sVEGFR-1. Oestrogen receptor status and VEGFR-2, Her-2/neu and TP level did not have a statistically significant effect on patient outcome in the univariate analyses.

When we assessed the prognostic value of angiogenesis-related factors in the subgroups divided by ER status, total VEGF was a significant prognostic factor for ER-positive group (*P*=0.0003) and not for ER-negative group (*P*=0.120) (Table 4 and Figure 1). In contrast, within the ER-negative group, sVEGFR-1 and the S/V ratio were found to be strong prognostic indicators (*P*=0.001 and 0.0001, respectively), but this was not the case for the ER-positive group. In particular, all patients with ER-negative and low S/V ratio tumours relapsed within 4 years after surgery (Figure 1). There was also a statistically significant benefit for OS with high sVEGFR-1 or a high S/V ratio for the ER-negative group (*P*=0.03 and 0.0002, respectively), but not for the ER-positive group. Figure 1 shows the DFS curves for total VEGF, sVEGFR-1 and the S/V ratio for the ER-positive population (*n*=111) and ER-negative subpopulation (*n*=75). The results of other subgroup analyses divided by total VEGF status, TP status and Her-2/neu status are summarised in Table 4.

All tumour- and clinico-pathological-related parameters regardless of whether they were statistically significant as seen by univariate analysis were included in the multivariate analysis. Variables showing statistically significant effects were retained and the others were dropped. The resulting multivariate analysis revealed that DFS and OS were improved in patients with low total VEGF (*P*<0.001 and *P*=0.043), a high S/V ratio (*P*=0.002 and 0.003), pathological low grade (*P*=0.015 and 0.034), tumour less than 5 cm in size (*P*=0.002 and 0.038) and negative nodal involvement (*P*=0.001 and 0.002, respectively) (Table 5). Soluble VEGFR-1 alone did not result in improved DFS or OS.

## DISCUSSION

Soluble VEGFR1 levels are frequently elevated in human breast cancer tissues. Out of 202 tumours, 155 contained higher concentrations of sVEGFR-1 than those of total VEGF. A recent study of the relationship between circulating sVEGFR-1 levels and preeclampsia reported that increased sVEGFR-1 is significantly associated with the development of preeclampsia, suggesting that immunodetectable sVEGFR-1 is biologically active (Levine *et al*, 2004). Regarding malignancies such as brain tumours or leukaemia, it was also documented that intratumoral or plasma sVEGFR-1 level is related to tumour phenotype or prognosis, suggesting that sVEGFR-1 plays a significant biological role not only during development or pregnancy but also in neoplasms (Lamszus *et al*, 2003; Hu *et al*, 2004).

Since simultaneous measurement of sVEGFR-1 and VEGFR-1 in the same sample is technically difficult, we were not able to compare the concentrations of sVEGFR-1 with those of VEGFR-1 directly. Nevertheless, as a preliminary study, we examined sVEGFR-1 and VEGFR-1 expressions in human umbilical vein endothelial cells (HUVECs) and in primary breast tumour tissues by Western blot using different types of lysis buffers with or without detergent. Using HUVECs as a positive control for both VEGFR-1 and sVEGFR-1, we confirmed that in the protein extract prepared without detergent, only sVEGFR-1 was detectable, and in contrast, in the protein extract prepared with detergent such as with RIPA buffer, both VEGFR-1 and sVEGFR-1 were detectable. We speculated that VEGFR-1 exists on a cell membrane, and the membrane fraction will not be lysed in a buffer without detergent. Then we examined expression of sVEGFR-1 and VEGFR-1 by Western blot analysis in 15 randomly selected primary breast cancer tissues prepared with different lysis buffers: 11 tumours had sVEGFR-1 expressions and its expressions were more dominant than VEGFR-1 expressions, and the concentration of sVEGFR-1 measured by ELISA was well correlated (data not shown). With samples treated without detergent, VEGFR-1 band was not detected in all cases. Although we have not examined all the cases that we used in the current study by Western blot analysis, the measurement of sVEGFR-1 expression with appropriate lysis buffer is meaningful with this ELISA system.

Total VEGF was determined to be a potent and independent prognostic indicator in both node-negative and node-positive cancers as reported in the previous studies (Toi *et al*, 2001; Ferrara *et al*, 2003). No significant prognostic value of free VEGF was observed in this study. It is difficult to explain why only total VEGF provides significant prognostic value but not free VEGF. Total VEGF concentration is a useful marker for predicting survival or disease progression. The current study using a highly sensitive sVEGFR-1 ELISA system confirmed that sVEGFR-1 is a significant prognostic indicator. In particular, we discovered that low sVEGFR-1 was related to an unfavourable prognosis, which was a slightly different result from that we had in a previous study with a relatively low-sensitivity sVEGFR-1 ELISA system, where we found a prognostic value of high sVEGFR-1 concentrations for favourable prognosis (Toi *et al*, 2002). The significance of the ratio of sVEGFR-1 to total VEGF (S/V ratio) as a prognostic marker was reconfirmed in this larger size of analysis. Particularly, a low S/V ratio was associated with unfavourable prognosis. Since sVEGFR-1 protein is estimated to be produced by both tumour cells and stromal cells in breast cancer microenvironment, it would be important to analyse the regulatory mechanisms of this balance more thoroughly.

In the subgroup analyses, we found that low sVEGFR-1 expression was significantly related to poor prognosis for ER-negative subgroup but not for ER-positive subgroup. The prognostic value of S/V ratio also significantly associated with ER negativity, and the correlation was more relevant than sVEGFR-1 status alone. It is reported that total VEGF expression is related to a poor prognosis in ER-positive patients rather than ER-negative patients (Linderholm *et al*, 2000; Foekens *et al*, 2001; Buteau-Lozano *et al*, 2002; Manders *et al*, 2003). Therefore, it is important to consider why the prognostic value of sVEGFR-1 or S/V ratio was associated with ER-negative status. Several explanations might be possible. First, the sensitivity of endothelial cells to VEGF might be different between ER-positive tumours and ER-negative tumours. Several reports discussed that ER-positive and ER-negative tumours display remarkably different gene expression phenotypes (Gruvberger *et al*, 2001). Also, ER-negative tumour cells produced larger amounts of growth factors and cytokines that can stimulate various types of cells including endothelial cells (Bando *et al*, 2003). Second, adjuvant therapies, especially postoperative hormone therapy, might cause a difference in the survival analysis between ER-positive and ER-negative subgroups. It is known that hormone treatments such as tamoxifen can downregulate VEGF expression in hormone-sensitive breast tumour cells or tumour tissues (Garvin and Dabrosin, 2003). In addition, it was recently documented that sVEGFR-1 was inducible in normal breast cell line and human breast cancer cell line, MCF-7, with hormone-dependent property in response to anti-oestrogen treatments (Elkin *et al*, 2004). In those cells, oestrogen was a potent downregulator of sVEGFR-1, and ER antagonism blocked the action dramatically. In the present study using nontreated primary tumours, sVEGFR-1 levels were significantly higher in PgR-negative tumours rather than PgR-positive tumours, which seems to support the idea that sVEGFR-1 is downregulated in purely hormone-dependent tumours such as ER-positive and PgR-positive tumours. According to these data, it might be possible to hypothesise that adjuvant hormonal treatments may suppress disease progression in patients having low S/V ratio, which is basically associated with poor survival, by modulating those expressions. In ER-negative patients, theoretically it does not happen. Many other possibilities could be raised to explain this translational research question.

Another important finding observed in subgroup analyses is that the total VEGF and sVEGFR-1 levels as well as the S/V ratio exhibited significant prognostic importance for low Her-2/neu tumours but not for high Her-2/neu tumours (Table 4). Transfection of the Her-2/neu gene enhances VEGF expression in breast cancer experimental models (Yen *et al*, 2000). In this study, the intratumoral concentrations of Her-2/neu were significantly correlated with those of VEGF. The regulatory mechanism of VEGF and sVEGFR-1 expressions could be different between Her-2/neu-positive and Her-2/neu-negative subgroups.

As to the regulatory mechanism of sVEGFR-1, several factors such as growth mediators and hypoxia are reported to induce the expression of VEGFR-1 and sVEGFR-1 in endothelial cells (Barleon *et al*, 1997). Among these inducers, hypoxia might be a key factor especially, because it is capable of regulating the expressions of multiple angiogenesis-related molecules simultaneously (Griffiths *et al*, 1997; Bando *et al*, 2003). It was found in this study that the intratumoral concentration of sVEGFR-1 significantly correlated with those of VEGFR-2, total VEGF, free VEGF and TP. Therefore, it is interesting to know the relationship with the markers of hypoxia in future analysis, and to understand the machinery of alternative splicing of sVEGFR-1 in both tumour-associated stromal cells and hormone-dependent cancer cells.

In conclusion, the intratumoral concentration of sVEGFR-1 and VEGF and the ratio of sVEGFR-1 to total VEGF were potent prognostic indicators in 202 primary breast tumours in our study. The expression level and the balance between VEGF and sVEGFR-1 molecules were thought to be important to understand the hormone dependency of breast cancer and the sensitivity or resistance to hormonal therapy. Recently, it was clinically demonstrated that anti-VEGF therapy brings survival benefit to cancer patients in colorectal cancer patients (Hurwitz *et al*, 2004). We speculate that the determination of VEGF and sVEGFR-1 will also be useful to distinguish anti-VEGF therapy-sensitive tumours from less sensitive tumours. Eventually, the quantification of VEGF and its related molecules will be important for understanding the tumour growth machinery, the disease progression and the survival prediction of primary breast cancer and for considering treatment strategies with hormonal therapies and antiangiogenesis therapies.

## Figures and Tables

**Table 1:** Patients characteristics

	**Number of patients (%)**
Patients enrolled	202
Median age (years)	55, range 30–86
Menopausal status	
Pre	86 (42.6)
Post	116 (57.4)
Tumour size (cm)	
<2	26 (12.9)
2–5	125 (61.9)
>5	51 (25.2)
Nodal involvement	
−	94 (46.5)
+	108 (53.5)
ER	
Positive	110 (54.5)
Negative	92 (45.5)
PgR	
Positive	105 (52.0)
Negative	97 (48.0)
Hormonal receptor	
ER+ and PR+	79 (39.1)
ER+ or PR+	56 (27.7)
ER− and PR−	67 (33.2)
Nuclear grade	
1	39 (19.3)
2	99 (49.0)
3	64 (31.7)
Recurrence	
+	57 (28.2)
−	145 (71.8)
Adjuvant therapy	
CAF (CEF)	54 (26.7)
CMF (CF)	18 (8.9)
FU derivatives	38 (18.8)
Tamoxifen	119 (58.9)
LH-RH	10 (5.0)

ER=oestrogen receptor; PgR=progesterone receptor; CAF (CEF)=cyclophosphamide, adriamycin or epirubicin and 5-FU; CMF (CF)=cyclophosphamide, methotrexate and 5-FU.

**Table 2:** Quantitation of total and free VEGF, sVEGFR-1, VEGFR-2, Her-2/neu and TP proteins in primary breast cancer tumour cytosol

		**Total VEGF**			**Free VEGF**			**sVEGFR-1**			**VEGFR-2**			**TP**			**Her-2/neu**		
	**No.**	**Mean**	**95% CI**	***P***	**Mean**	**95% CI**	***P***	**Mean**	**95% CI**	***P***	**Mean**	**95% CI**	***P***	**Mean**	**95% CI**	***P***	**Mean**	**95% CI**	***P***
Patients enrolled	202	0.532	0.432–0.632		0.135	0.118–0.152		0.949	0.849–1.048		0.105	0.098–0.112		194.9	181.1–209.9		13.057	10.147–15.867	
Menopause																			
Pre	86	0.534	0.420–0.644	NS	0.132	0.106–0.170	NS	0.896	0.740–1.053	NS	0.102	0.087–0.131	NS	194.2	177.9–219.1	NS	15.265	10.189–20.340	NS
Post	116	0.530	0.416–0.644		0.141	0.113–0.156		0.994	0.858–1.130		0.108	0.098–0.123		195.3	172.8–213.4		11.244	8.005–14.483	
Tumour size																			
T1	26	0.532	0.420–0.644	NS	0.102	0.075–0.128	NS	0.917	0.737–1.97	NS	0.103	0.089–0.117	NS	196.7	166.3–227.1	NS	11.916	5.163–18.669	
T2	125	0.536	0.425–0.647		0.141	0.115–0.168		0.962	0.823–1.097		0.107	0.095–0.119		198.6	178.7–218.4		10.785	7.563–14.007	
T3 or more	51	0.520	0.356–0.684		0.136	0.117–0.155		0.956	0.714–1.198		0.105	0.085–0.125		187.0	155.6–218.5		19.290	12.006–26.575	0.01^a^
Nodal status																			
n−	94	0.550	0.462–0.638	NS	0.139	0.116–0.163	NS	0.954	0.852–1.055	NS	0.106	0.096–0.116	NS	201.9	188.9–214.9	NS	11.983	8.285–15.682	NS
n+	108	0.516	0.392–0.640		0.133	0.102–0.163		0.899	0.751–1.047		0.104	0.091–0.117		187.6	166.5–208.5		13.860	9.568–18.152	
Hormonal receptor																			
ER+	120	0.458	0.401–0.515	0.042	0.119	0.098–0.140	0.032	0.905	0.784–1.027	NS	0.109	0.098–0.121	NS	201.0	183.6–218.4	NS	10.924	7.985–13.864	0.048
ER−	82	0.621	0.464–0.778		0.159	0.092–0.227		1.031	0.849–1.213		0.102	0.087–0.117		186.9	161.5–212.3		16.117	10.473–21.761	
PgR+	105	0.450	0.342–0.559	0.055	0.112	0.087–0.138	0.012	0.81	0.695–0.926	0.011	0.103	0.094–0.112	NS	190.2	181.0–210.5	NS	12.170	7.957–16.384	NS
PgR−	97	0.600	0.491–0.710		0.160	0.133–0.186		1.094	0.933–1.254		0.110	0.098–0.122		200.3	179.5–221.1		13.817	9.870–17.765	
Recurrence																			
+	57	0.658	0.516–0.800	NS	0.163	0.129–0.197	NS	0.942	0.741–1.144	NS	0.103	0.082–0.123	NS	203.6	169.8–237.7	NS	13.612	10.285–16.939	NS
−	145	0.482	0.3801–0.584		0.126	0.104–0.148		0.941	0.825–1.057		0.107	0.098–0.116		192.8	177.1–208.6		11.491	5.617–17.366	
Nuclear grade																			
1	39	0.531	0.450–0.612	NS	0.104	0069–0.138	NS	0.868	0.849–1.048	NS	0.110	0.091–0.129	NS	186.9	168.4–205.4	NS	12.965	6.758–19.172	
2	99	0.503	0.400–0.606		0.141	0.112–0.170		0.917	0.772–1.062		0.099	0.089–0.109		200.8	180.9–220.7		10.323	6.969–13.678	
3	64	0.578	0.468–0.688		0.148	0.116–0.180		1.075	0.864–1.286		0.110	0.095–0.125		205.6	178.4–232.8		16.947	10.680–23.214	0.032^b^

Intratumoral total and free VEGF, sVEGFR-1, VEGFR-2, TP, Her-2/neu, ER and PR protein levels were measured by quantitative ELISA and enzyme immunoassay (see ‘Materials and Methods’). The results reflect the mean values, 95% CI and *P*-value. Levels of ER and PgR more than 10 fmol mg^−1^ total protein were considered positive (+) and less than 5 fmol mg^−1^ total protein negative (−). The correlations between each biological factor and clinico-pathological parameters were analysed using Student's *t*-test. Differences at *P*<0.05 were considered to be statistically significant. NS=not significant.

a Statistically significant between T2 and T3.

b Statistically significant between nuclear grade 2 and 3.

**Table 3:** Univariate analysis of clinico-pathological and tumour biologic factors for DFS and OS

		**DFS**			**OS**		
**Parameter**	***n* (total 186)**	***P***	**Hazard ratio**	**95% CI**	***P***	**Hazard ratio**	**95% CI**
*Clinical features*							
ER							
Positive	111	0.503	0.835	0.493–1.415	0.098	0.604	0.332–1.099
Negative	75	Baseline			Baseline		
PgR							
Positive	92	0.051	0.584	0.340–1.003	0.017	0.461	0.243–0.872
Negative	94	Baseline			Baseline		
Nuclear grade							
1	33	0.002	0.111	0.026–0.466	0.011	0.075	0.010–0.556
2 and 3	153	Baseline			Baseline		
Tumour size (cm)							
1–5	146	0.0001	0.224	0.131–0.384	0.0002	0.313	0.170–0.579
5 or more	42	Baseline			Baseline		
Nodal involvement							
n+	104	Baseline			Baseline		
n−	84	0.0001	0.207	0.104–0.410	0.0005	0.271	0.130–0.565
Lymph vessel (ly) involvement							
ly positive	140	Baseline			Baseline		
ly negative	46	0.001	0.107	0.026–0.438	0.008	0.07	0.010–0.512
Blood vessel (v) involvement							
v positive	131	Baseline			Baseline		
v negative	55	0.016	0.396	0.187–0.842	0.05	0.421	0.177–1.002
							
*Biological features*							
Total VEGF							
High (>0.532 ng mg^−1^ protein)	64	0.002	2.231	1.306–3.811	0.006	2.278	1.233–4.206
Low (<0.532 ng mg^−1^ protein)	122	Baseline			Baseline		
Free VEGF							
High (>0.135 ng mg^−1^ protein)	71	0.047	1.72	0.999–2.962	0.204	1.488	0.803–2.758
Low (<0.135 ng mg^−1^ protein)	115	Baseline			Baseline		
SVEGFR-1							
High (>0.435 ng mg^−1^ protein)	29	0.040	0.526	0.282–0.983	0.05	0.527	0.258–1.075
Low (<0.435 ng mg^−1^ protein)	157	Baseline			Baseline		
VEGFR-2							
High (>0.105 ng mg^−1^ protein)	119	0.316	0.762	0.447–1.299	0.883	1.047	0.563–1.947
Low (<0.105 ng mg^−1^ protein)	67	Baseline			Baseline		
Her-2/neu							
High (>13.5 ng mg^−1^ protein)	41	Baseline			Baseline		
Low (<13.5 ng mg^−1^ protein)	145	0.362	1.394	0.682–2.848	0.753	0.889	0.426–1.854
TP							
High (>194.9 ng mg^−1^ protein)	97	0.785	0.928	0.541–1.590	0.955	0.983	0.533–1.813
Low (<194.9 ng mg^−1^ protein)	89	Baseline			Baseline		
S/V ratio							
High (⩾0.5)	170	0.008	0.368	0.186–0.731	0.0002	0.267	0.127–0.561
Low (<0.5)	16	Baseline			Baseline		

Prognostic parameters evaluated included ER and PR status (<10 fmol mg^−1^ protein *vs* more than 10 fmol mg^−1^ protein), primary tumour size, axillary lymph node involvement, total VEGF level, free VEGF level, sVEGFR-1 level, VEGFR-2 level, Her-2/neu protein level, TP level and S/V ratio (sVEGFR-1/total VEGF ratio). For total and free VEGF, VEGFR-2 and Her-2 protein levels, cutoff values were determined as mean values. For sVEGFR-1 and S/V ratio, cutoff values were determined according to a stepwise method (see Results). The median follow-up was 64 months. Survival analysis was performed on 186 cases excluding six patients with ductal carcinoma *in situ* and 10 patients who did not show up for the follow-up. The prognostic significance was assessed using the log-rank test. All *P*-values are two-sided. Hazard ratio indicated Cox model hazard ratio.

**Table 4:** Univariate subgroup analysis of tumour biologic factors for DFS and OS

	***P*-values**																					
	**Total case**		**ER+ cases**		**ER− cases**		**PgR+ cases**		**PgR− cases**		**n+ cases**		**n− cases**		**TP-high cases**		**TP-low cases**		**Her-2/neu-high cases**		**Her-2/neu-low cases**	
	**(186 cases)**		**(111 cases)**		**(75 cases)**		**(92 cases)**		**(94 cases)**		**(101 cases)**		**(85 cases)**		**(97 cases)**		**(89 cases)**		**(41 cases)**		**(145 cases)**	
	**54 relapse**		**27 relapse**		**27 relapse**		**20 relapse**		**34 relapse**		**44 relapse**		**10 relapse**		**27 relapse**		**27 relapse**		**9 relapse**		**45 relapse**	
	**DFS**	**OS**	**DFS**	**OS**	**DFS**	**OS**	**DFS**	**OS**	**DFS**	**OS**	**DFS**	**OS**	**DFS**	**OS**	**DFS**	**OS**	**DFS**	**OS**	**DFS**	**OS**	**DFS**	**OS**
Total VEGF	0.002	0.006	0.0003	0.003	0.120	0.091	0.038	0.114	0.058	0.062	0.012	0.050	0.002	0.006	0.001	0.002	0.242	0.479	0.341	0.575	0.002	0.009
Free VEGF	0.047	0.204	0.003	0.018	0.940	0.827	0.042	0.288	0.536	0.660	0.030	0.230	0.220	0.320	0.004	0.034	0.903	0.722	0.381	0.556	0.066	0.262
sVEGFR-1	0.040	0.050	0.750	0.702	0.001	0.033	0.354	0.538	0.028	0.039	0.328	0.663	0.847	0.071	0.224	0.275	0.050	0.168	0.349	0.744	0.028	0.035
VEGFR-2	0.316	0.883	0.680	0.713	0.210	0.506	0.317	0.650	0.773	0.918	0.628	0.227	0.162	0.078	0.204	0.796	0.987	0.907	0.237	0.142	0.826	0.315
S/V ratio (0.5)	0.008	0.0002	0.404	0.067	0.0001	0.0002	0.074	0.024	0.012	0.013	0.006	0.008	0.001	0.0001	0.004	0.007	0.012	0.012	0.057	0.530	0.0007	0.0001

The univariate analysis for DFS and OS was performed for subgroups determined by ER status (cutoff value 10 fmol mg^−1^ protein), PgR status (cutoff value 10 fmol mg^−1^ protein), node involvement (positive or negative), TP expression level (cutoff value 194.9 ng mg^−1^ protein) and Her-2/neu expression level (cutoff value 13.5 ng mg^−1^ protein). The cutoff values for total VEGF, free VEGF, sVEGFR-1, VEGFR-2 and S/V ratio were 0.532 ng mg^−1^ protein, 0.135 ng mg^−1^ protein, 0.435 ng mg^−1^ protein, 0.105 ng mg^−1^ protein and 0.5, respectively. The listed values are *P*-values calculated using the log-rank test. All *P*-values are two-sided.

**Table 5:** Multivariate analysis of clinico-pathological and tumour biologic factors for DFS and OS

	**DFS**			**OS**		
**Parameter**	**Hazard ratio**	**95% CI**	***P***	**Hazard ratio**	**95% CI**	***P***
Nuclear grade 1	0.172	0.041–0.598	0.015	0.132	0.016–0.861	0.034
Tumour size <5 cm	0.391	0.215–0.713	0.002	0.502	0.261–0.965	0.038
Node negative	0.269	0.121–0.598	0.001	0.3	0.136–0.656	0.002
VEGF-A (<0.532 ng mg^−1^ protein)	0.458	0.257–0.814	<0.001	0.499	0.256–0.972	0.043
sVEGFR-1/total VEGF-A >0.5	0.225	0.107–0.472	0.002	0.295	0.131–0.666	0.003

Hazard ratio indicates Cox model proportional hazard ratio; *P* is the Wald model *P*-value.
